# Third-Trimester Maternal and Neonatal Cord Blood Vitamin D3 Levels in Urban South India: An Observational Cross-Sectional Study

**DOI:** 10.7759/cureus.96845

**Published:** 2025-11-14

**Authors:** Amulya V, Vinayaka G, Vikram Sakaleshpur Kumar, Vijayasuryakiran KM, Ganesh S Mena, Samadhara V Jogi, Nimishashree K Srinivas

**Affiliations:** 1 Pediatrics, Subbaiah Institute of Medical Sciences and Research Center, Shivamogga, IND; 2 Pediatrics and Child Health, Subbaiah Institute of Medical Sciences and Research Center, Shivamogga, IND; 3 Pediatric Medicine, Sarji Maternal and Child Hospital, Shivamogga, IND

**Keywords:** correlation, maternal health, neonatal outcomes, nutritional status, vitamin d deficiency

## Abstract

Introduction: Vitamin D plays a crucial role in bone health, immune function, and metabolic regulation. Despite ample sunlight, vitamin D deficiency (VDD) remains prevalent in India, particularly among pregnant women and neonates. This study investigates the correlation between maternal serum and neonatal cord blood vitamin D3 levels during the third trimester and at birth, assessing how maternal vitamin D status influences neonatal outcomes.

Objective: This research aims to study the correlation between maternal serum vitamin D3 levels in the third trimester and neonatal cord blood vitamin D3 levels.

Methods: This observational cross-sectional study was conducted at a tertiary care center from August 2022 to February 2024. The study included 58 pregnant women aged 18-45 and their neonates. Maternal serum samples were collected during the third trimester, and cord blood samples were obtained at delivery. Data were analyzed using Statistical Package for the Social Sciences (SPSS) 23.0 (IBM Corp., Armonk, NY), with categorical variables expressed as frequencies/percentages and numerical data as mean ± standard deviation (SD). Pearson's correlation assessed the maternal-neonatal vitamin D3 relationship.

Results: A strong positive correlation was found between maternal and neonatal vitamin D3 levels (Pearson's coefficient: 0.74; p < 0.001; 95% confidence interval {CI}: 0.62-0.83). Among neonates born to vitamin D-deficient mothers, 55.6% were also deficient. No significant associations were observed between maternal vitamin D3 levels and demographic factors (age, religion, education, and socioeconomic status {SES}; p > 0.05) or neonatal outcomes (birth weight, length, and head circumference). Limitations include an exclusive focus on urban residents and potential recall bias in self-reported supplementation data.

Conclusion: Maternal vitamin D levels strongly correlate with neonatal levels, emphasizing the need for routine prenatal screening and targeted supplementation to address deficiencies and improve maternal and neonatal health outcomes.

## Introduction

Vitamin D is not just the bone-associated "sunshine" vitamin but also a metabolic modulator, almost similar to a hormone. Its primary source is cutaneous synthesis in the skin, facilitated by ultraviolet B (UVB) rays, fulfilling about 90% of the body's requirement [1,2]. Additionally, dietary sources such as egg yolks, fatty fish, and fortified foods contribute to vitamin D intake [3]. Rickets, a disease of growing bones, is attributable to vitamin D deficiency (VDD) and is preventable with adequate nutritional intake of vitamin D [4,5]. Other associations of VDD include type 2 diabetes, cardiovascular dysfunction, and autoimmune diseases during later life [6]. Vitamin D deficiency is a major public health concern in India, with community-based studies over the past decade reporting a prevalence of 50%-94% in healthy individuals. These studies, covering various age groups, highlight the issue's magnitude [7]. Recent data show that VDD prevalence among pregnant women worldwide ranges from 45% to 100% and, in India, from 42% to 93%, with neonates often mirroring this deficiency [8,9]. VDD affects both developed and developing countries. In developed regions such as Europe and North America, limited sunlight, especially in higher latitudes and winter, along with lifestyle and dietary habits, contributes to low vitamin D levels [6]. In developing countries such as India, despite ample sunlight, reduced outdoor activity, dietary habits, and cultural practices lead to widespread deficiency, particularly in pregnant women and neonates [10]. Addressing this requires standardized supplementation guidelines, food fortification, increased awareness, and public health campaigns promoting safe sun exposure [11]. Furthermore, the extent of vitamin D deficiency often differs between urban and rural populations due to variations in sunlight exposure, dietary diversity, and outdoor activity. While urbanization tends to reduce natural sun exposure, rural populations may benefit from higher UVB exposure, leading to lower deficiency prevalence. In addition, emerging research indicates that the altered expression of vitamin D receptors (VDRs) during pregnancy may influence transplacental vitamin D transfer and pregnancy outcomes such as preeclampsia and gestational diabetes, warranting further investigation.

While vitamin D is primarily associated with bone health, it also serves as a vital metabolic modulator, impacting various physiological functions [12,13]. Recent studies have highlighted significant deficiencies among pregnant women and newborns, yet several critical issues remain underexplored. Studies have also indicated altered expression patterns of vitamin D receptors (VDRs) and related proteins in pregnancy complications such as preeclampsia, suggesting broader implications for maternal and fetal health [14]. Vitamin D deficiency during pregnancy has been linked to a higher risk of complications such as pregnancy-induced hypertension, gestational diabetes, miscarriage, preterm delivery, low birth weight, and skeletal or respiratory disorders in the newborn. These adverse outcomes are often attributed to inadequate sunlight exposure or poor nutritional intake, with fetal serum 25-hydroxy vitamin D levels being entirely reliant on maternal stores [15,16]. Specifically, the relationship between maternal and neonatal vitamin D levels, whether neonates deplete maternal stores or mothers become deficient while maintaining neonatal sufficiency, remains unclear. Additionally, no standardized reference for cord blood vitamin D exists, despite its reliability for transplacental transfer assessment. Cord blood sampling is particularly advantageous as it is minimally invasive and allows for early intervention. This research aims to fill these gaps by examining the correlation between third-trimester maternal and cord blood vitamin D3 levels. Given the high prevalence of VDD and its association with adverse outcomes such as low birth weight, impaired neonatal development, and skeletal disorders, this study provides essential insights into the transplacental transfer of vitamin D [17-19].

This study explores the challenges of ensuring adequate vitamin D supplementation during pregnancy, considering absorption variations and sun exposure. The lack of large-scale urban studies and national supplementation guidelines is a key gap in public health research, especially in India, where VDD is widespread among pregnant women and neonates. Given its high burden and link to adverse pregnancy outcomes, this study examines the correlation between third-trimester maternal and cord blood vitamin D3 levels in urban populations, where VDD is particularly prevalent. By addressing these gaps, it aims to provide insights to guide future maternal and neonatal vitamin D supplementation guidelines in India.

## Materials and methods

This observational cross-sectional study was conducted at a tertiary care center from August 2022 to February 2024, following ethical approval from the Institutional Ethics Committee of Subbaiah Institute of Medical Sciences (approval number: IEC-SUIMS/23/JULY/22). A total of 58 pregnant women, aged 18-45 years, with no known morbidity, and their neonates were included after providing written consent. Maternal samples were collected between 32 and 38 weeks of gestation, aligning with the third-trimester window most representative of late-pregnancy vitamin D status. Early preterm neonates were excluded due to their established higher risk of vitamin D deficiency. Early preterm neonates were excluded because their vitamin D stores and calcium metabolism differ substantially from term neonates. Although this exclusion minimized confounding, it also limits the applicability of our results to preterm infants, who remain at higher risk of deficiency.

The sample size was determined based on a power calculation, considering a standard deviation (SD) of 11.1 in cord blood vitamin D levels and 7.8 in maternal blood levels and an expected mean difference of 7.5 [20]. With 90% power and a 1% alpha error, the minimum required sample size was 49, and a final sample size of 58 was chosen. Maternal blood (2 mL) was collected during the third trimester at the time of delivery, coinciding with routine blood draws, and 2 mL of cord blood was also collected. The VIDAS® 25 OH Vitamin D TOTAL (VITD) Kit (bioMérieux, Marcy-l'Étoile, France), based on an enzyme immunoassay competition principle with final fluorescent detection (enzyme-linked fluorescent assay {ELFA}), was used to analyze vitamin D levels. Reagents were prepared according to the manufacturer's instructions; the assay process took approximately 40 minutes. The thresholds for vitamin D levels were based on Indian standards, specifically the Indian Academy of Pediatrics guidelines for neonates and the Endocrine Society of India guidelines for pregnant women [18,19]. To address potential confounders, adjustments were made for maternal BMI and gestational age in the statistical models. Specifically, multivariable regression models were employed to ensure these factors did not bias the results. Missing data were handled by excluding incomplete samples or data points, and imputation was used when necessary for small amounts of missing data. Maternal vitamin D supplementation was recorded through self-reports. To mitigate recall bias, medical records were reviewed whenever available to verify reported supplementation status. Statistical analyses included correlation analyses to ensure that findings were robust and accounted for potential confounders.

## Results

The statistical analysis was conducted using Statistical Package for the Social Sciences (SPSS) 23.0 (IBM Corp., Armonk, NY). Categorical variables were represented as frequencies and percentages, while numerical data were expressed as mean and standard deviation (SD). The chi-square (χ²) test was employed for categorical data analysis, with a p-value of less than 0.05 considered statistically significant. Pearson's correlation coefficient was used to evaluate linear relationships, with values closer to 1 indicating a stronger positive correlation.

The study analyzed maternal vitamin D3 levels across demographic and socioeconomic factors (Table 1). No significant associations were found, but trends suggested higher deficiency among younger women (20-29 years). Though vitamin D3 intake and socioeconomic status (SES) lacked statistical significance, their distribution suggests potential clinical relevance. Religion was analyzed for cultural or dietary influences but showed no significant associations (χ² = 5.151; p = 0.272). Other variables, including education (χ² = 4.036; p = 0.133), socioeconomic status (χ² = 3.123; p = 0.537), gender (χ² = 0.11; p = 0.946), and urban residence, also showed no significance. Neonatal characteristics and vitamin D3 levels were examined (Table 2). No significant association was found with preterm birth (χ² = 2.835; p = 0.242) or birth weight (p = 0.768). Growth parameters, including length (χ² = 4.814; p = 0.307) and head circumference (χ² = 2.305, p = 0.68), showed no significant differences.

**Table 1:** Maternal Characteristics and Serum Vitamin D3 Levels Maternal demographic and socioeconomic characteristics in relation to serum vitamin D3 levels. Categorical variables are presented as frequencies and percentages. Statistical analysis was performed using the chi-square (χ²) test. P < 0.05 was considered statistically significant p: probability value

Maternal Characteristics	Vitamin D3 Levels			Chi-Square Value	P-value
	Deficient (<20 ng/mL) (n = 36)	Insufficient (20-30 ng/mL) (n = 17)	Sufficient (>30 ng/mL) (n = 5)		
Age					
20-29 Years	30 (83.3%)	12 (70.6%)	5 (100%)	χ² = 2.501	p = 0.286
30-39 Years	6 (16.7%)	5 (29.4%)	0 (0%)		
Religion					
Christian	0 (0.0%)	1 (5.9%)	0 (0.0%)	χ² = 5.151	p = 0.272
Hindu	23 (59%)	11 (28.2%)	5 (12.8%)		
Muslim	13 (72.2%)	5 (27.8%)	0 (0.0%)		
Area of Residence					
Urban	36 (100%)	17(100%)	5 (100%)	-	-
Educational Qualification					
Graduate	19 (52.8%)	10 (58.8%)	5 (100%)	χ² = 4.036	p = 0.133
High School	17 (47.2%)	7 (41.2%)	0 (100%)		
Socioeconomic Status					
Class 2	11 (30.6%)	4 (23.5%)	3 (60%)	χ² = 3.123	p = 0.537
Class 3	16 (44.4%)	8 (47.1%)	2 (40%)		
Class 4	9 (25%)	5 (29.4%)	0 (0%)		
Vitamin D3 Supplementation					
Yes	28 (77.8%)	15 (88.2%)	3 (60%)	χ² =2.013	p = 0.365
No	8 (22.2%)	2 (11.8%)	2 (40%)		

**Table 2:** Neonatal Characteristics and Serum Vitamin D3 Levels Neonatal characteristics and growth parameters in relation to serum vitamin D3 levels. Categorical variables are presented as frequencies and percentages. Statistical analysis was performed using the chi-square (χ²) test. P < 0.05 was considered statistically significant p, probability value; AGA, appropriate for gestational age; SGA, small for gestational age; LGA, large for gestational age

Neonatal Characteristics	Vitamin D3 Levels			Chi-Square Value	P-value
	Deficient (<12 ng/mL) (n = 36)	Insufficient (12-20 ng/mL) (n = 17)	Sufficient (>20 ng/mL) (n = 5)		
Gender					
Male	11 (47.8%)	10 (50%)	8 (53.3%)	χ² = 0.11	p = 0.946
Female	12 (52.2%)	10 (50%)	7 (46.7%)		
Period of Gestation					
Preterm	0 (0.0%)	1 (5.9%)	0 (0.0%)	χ² = 2.835	p = 0.242
Term	21 (91.3%)	18 (90%)	11 (73.3%)		
Birth Weight					
<2.5 kg	4 (17.4%)	4 (20%)	2 (13.3%)	χ² = 1.826	p = 0.768
2.5-3.5 kg	18 (78.3%)	16 (80%)	13 (86.7%)		
>3.5 kg	1 (4.3%)	0 (0%)	0 (0%)		
Length					
<10th Centile	2 (8.7%)	0 (0%)	0 (0%)	χ² = 4.814	p = 0.307
10th-90th Centile	20 (87%)	20 (100%)	15 (100%)		
>90th Centile	1 (4.3%)	0 (0%)	0 (0%)		
Head Circumference					
<10th Centile	1 (4.3%)	1 (5%)	0 (0%)	χ² = 2.305	p = 0.68
10th-90th Centile	21 (91.3%)	19 (95%)	15 (100%)		
>90th Centile	1 (4.3%)	0 (0%)	0 (0%)		
Appropriateness for Gestational Age					
AGA	17 (73.9%)	16 (80%)	14 (93.3%)	χ² = 3.279	p = 0.512
LGA	1 (4.3%)	0 (0%)	0 (0%)		
SGA	5 (21.7%)	4 (20%)	1 (6.7%)		
Maternal Vitamin D3 Levels					
Deficient	20 (55.6%)	14 (38.9%)	2 (5.6%)	χ² = 27.604	p < 0.001
Insufficient	3 (17.6%)	6 (35.3%)	8 (47.1%)		
Sufficient	0 (0%)	0 (0%)	5 (100%)		

**Figure 1:**
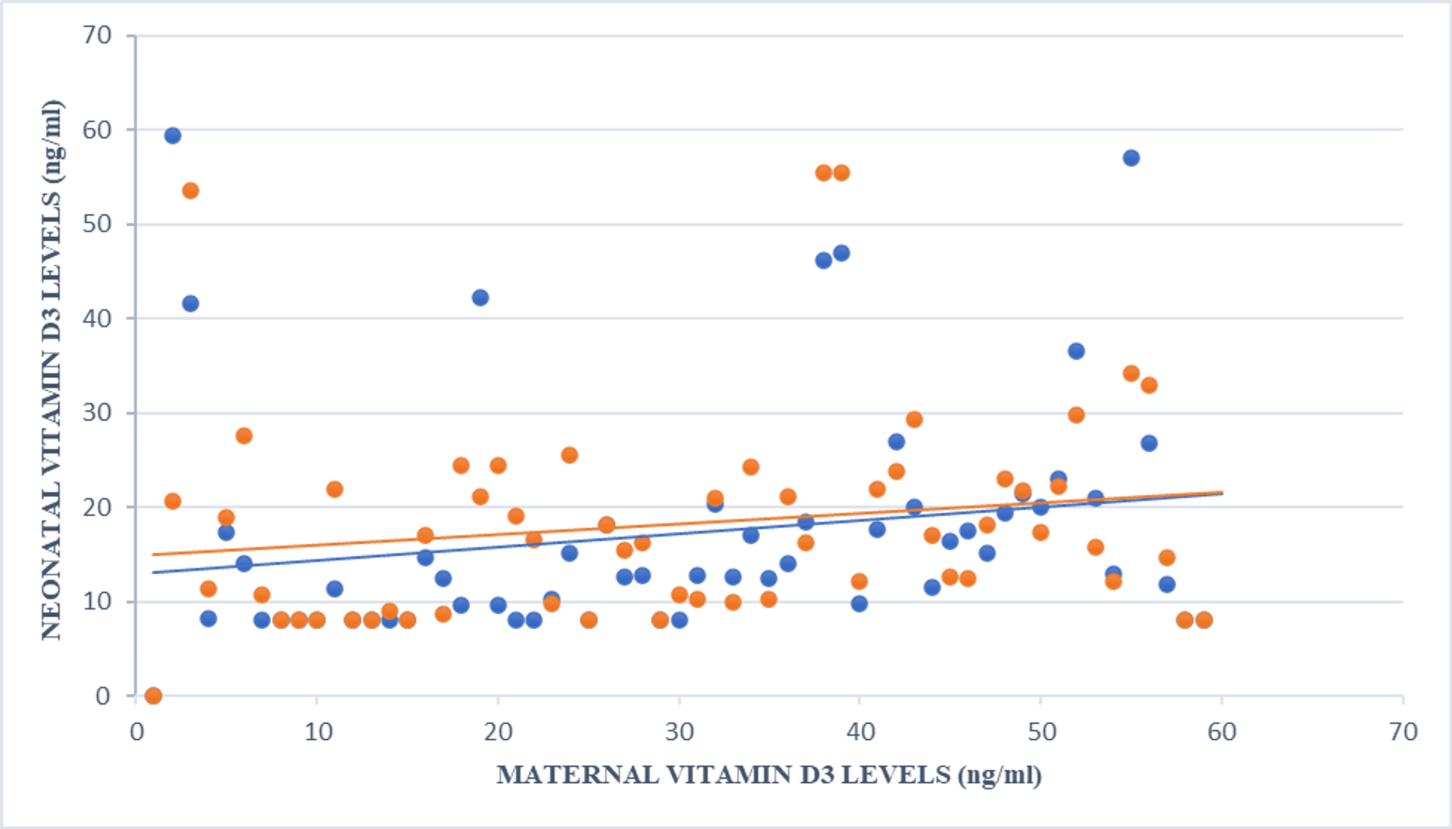
Scatter Plot of Maternal Vitamin D3 Levels Versus Neonatal Vitamin D3 Levels Scatter plot showing the correlation between maternal serum vitamin D3 levels and neonatal cord blood vitamin D3 levels (r = 0.74; 95% confidence interval {CI}: 0.62-0.83; p < 0.001). Statistical significance was defined as p < 0.05

A strong correlation was observed between maternal and neonatal vitamin D3 levels (Figure 1), with 55.6% of neonates born to deficient mothers also deficient, while none of the neonates of sufficient mothers had deficiency. Pearson's correlation confirmed a strong linear relationship (r = 0.74; 95% confidence interval {CI}: 0.62-0.83), with chi-square analysis showing significance (χ² = 42.3; p < 0.001). These findings highlight the importance of maintaining adequate maternal vitamin D levels for neonatal health, essential for bone development and immune function. Descriptive statistics showed an average neonatal birth weight of 2.864 kg, a length of 48.905 cm, a head circumference of 34.017 cm, and a mean neonatal vitamin D3 level of 15.443, compared to a maternal mean of 17.322.

## Discussion

Our study has highlighted a strong and statistically significant correlation between maternal and neonatal vitamin D3 levels (Pearson's correlation: 0.74; p < 0.001; 95% CI: 0.62-0.83), confirming the crucial role maternal vitamin D status plays in determining neonatal vitamin D levels. Specifically, 55.6% of neonates born to mothers with deficient vitamin D3 levels were also found to be deficient, while those born to mothers with sufficient levels exhibited a 100% sufficiency rate. This aligns with previous studies by Sathish et al. (2017) [20] and Avinash Sangle et al. (2020) [21], which reported significant correlations. However, Rai et al. (2020) found no significant correlation, highlighting variability due to geographic, demographic, and methodological differences [22]. Similarly, Kochar et al. (2019) reported that 73% of neonates had deficient cord blood levels, paralleling maternal deficiency, and identified significant associations with birth weight, indicating clinical implications of suboptimal prenatal vitamin D status [23].

Beyond the direct correlation, our findings examined demographic factors influencing maternal vitamin D3 levels. The high prevalence of deficiency across age, religion, residence, education, and socioeconomic status (SES) indicates widespread deficiency. Among mothers aged 20-29 years, 83.3% were deficient, aligning with Sachan et al.'s study (2005) [9]. While 72.2% of deficient mothers were Muslims, no significant association emerged (p = 0.272), suggesting shared cultural practices. Similarly, education (p = 0.133) and SES (p = 0.537) showed no significant correlation, reinforcing findings that factors such as limited sun exposure and uniform dietary habits contribute more to deficiency than economic status. Marwaha et al. (2011) found no significant variation across various religious groups, supporting widespread deficiency across demographics [24]. The lack of significant association between religion and vitamin D levels likely reflects homogeneous lifestyle and dietary patterns across faiths in this urban population, where sun exposure and clothing habits are similar, irrespective of religion.

Furthermore, the study strengthens the relevance of cord blood analysis as a reliable and minimally invasive indicator of neonatal vitamin D status. High rates of deficiency in cord blood samples in the context of maternal deficiency suggest that monitoring these levels could help inform neonatal supplementation needs. Findings by Anbalagan et al. (2021) similarly support the clinical utility of cord blood measurements, particularly in predicting deficiency among neonates with low birth weights [25].

The study also examined several neonatal variables, including gender, gestational age, birth weight, length, head circumference, and appropriateness for gestational age (appropriate for gestational age {AGA}, small for gestational age {SGA}, and large for gestational age {LGA}), in relation to neonatal vitamin D3 levels. No significant associations were found between these variables and vitamin D3 status (p > 0.05). For instance, gender distribution was nearly equal among neonates with deficient, insufficient, and sufficient vitamin D3 levels (p = 0.946), aligning with findings by Kumar et al. (2020), who also reported no gender-based differences in neonatal vitamin D levels [6]. Similarly, gestational age did not significantly influence vitamin D3 levels, with 91.3% of deficient neonates being term (p = 0.242), consistent with Sathish et al. (2017), who found no correlation between gestational age and vitamin D status [20]. Birth weight, length, and head circumference also showed no significant associations, echoing results from Aji et al. (2020), who noted that anthropometric measures were unaffected by vitamin D levels [26]. The lack of association with AGA/SGA/LGA categories (p = 0.512) further supports the conclusion that neonatal growth parameters may be influenced more by maternal and environmental factors than by vitamin D3 status alone [20]. These findings underscore the complexity of neonatal vitamin D dynamics and highlight the need for further research to elucidate underlying mechanisms.

The clinical implications are significant. Given the strong maternal-neonatal correlation, routine maternal screening and supplementation during pregnancy could help mitigate neonatal vitamin D deficiency. These findings support integrating standardized screening and fortification programs into antenatal care. Public health interventions, including targeted supplementation programs and awareness campaigns, are necessary to address this widespread issue. Additionally, educating healthcare providers and expectant mothers on the importance of vitamin D can play a pivotal role in reducing deficiency rates.

Several study limitations must be acknowledged. As this study exclusively included urban participants, the findings may not be fully generalizable to rural populations where outdoor activity and sunlight exposure differ significantly. Future multicentric studies including rural cohorts are warranted to evaluate environmental and lifestyle influences on maternal and neonatal vitamin D levels. Urban lifestyles reduce sun exposure, impacting vitamin D levels. Seasonal variations in vitamin D synthesis were not accounted for, and self-reported supplementation data introduced recall bias, impacting accuracy. Future studies should include larger, more diverse populations for better generalizability. As this was a cross-sectional analysis, it identifies associations but not causality; however, the correlation observed remains statistically robust and clinically meaningful within this design framework. A notable limitation is the absence of universally standardized reference ranges for cord blood vitamin D levels. Our analysis used Indian Academy of Pediatrics and Endocrine Society of India thresholds, but future large-scale studies are needed to establish global norms for neonatal vitamin D sufficiency.

Notably, no significant association was found between maternal vitamin D levels and neonatal outcomes such as birth weight or growth parameters. Since fetal growth is multifactorial, long-term vitamin D effects may be more relevant than immediate birth outcomes. While maternal vitamin D levels emerged as the strongest determinant of neonatal vitamin D status, multifactorial influences such as genetics, dietary intake, and sunlight exposure may also play contributory roles. Follow-up studies are essential to assess postnatal growth and child development. Addressing these gaps can refine maternal vitamin D guidelines and improve neonatal health strategies in India.

## Conclusions

This study establishes a significant and clinically relevant correlation between maternal and neonatal vitamin D3 levels, reinforcing the role of maternal status as the primary determinant of neonatal sufficiency. All neonates born to vitamin D-sufficient mothers were themselves sufficient, with a strong correlation coefficient (r = 0.74; p < 0.001; 95% CI: 0.62-0.83), underscoring the effectiveness of maternal-fetal transfer. The widespread burden of vitamin D deficiency in both mothers and neonates underscores the need for strengthening routine screening and individualized supplementation strategies during pregnancy.

Importantly, the disconnect between reported supplementation and maternal vitamin D levels exposes the limitations of generalized strategies currently in place. To address this, trimester-specific screening should be integrated into national antenatal care programs, enabling individualized, evidence-based supplementation protocols. Public health campaigns focusing on high-risk groups, particularly urban populations with low sun exposure, are essential to bridge the awareness and implementation gap. This study contributes uniquely to existing literature by providing region-specific data from South India and directly correlating third-trimester maternal and cord blood vitamin D3 levels. These findings offer a practical framework for refining maternal-child nutrition policies and advancing targeted interventions to reduce the burden of vitamin D deficiency in India.
